# Cornified Epithelial Teeth of Jawless Vertebrates Contain Proteins Similar to Keratin-Associated Proteins of Mammalian Skin Appendages

**DOI:** 10.3390/jdb13020018

**Published:** 2025-05-19

**Authors:** Attila Placido Sachslehner, David A. D. Parry, Leopold Eckhart

**Affiliations:** 1 Department of Dermatology, Medical University of Vienna, 1090 Vienna, Austria; attila.sachslehner@meduniwien.ac.at; 2 School of Natural Sciences, Massey University, Palmerston North 4442, New Zealand; d.parry@massey.ac.nz

**Keywords:** keratin, keratin-associated protein, keratinocytes, convergent evolution, differentiation, teeth, cornification, lamprey, cyclostomes

## Abstract

Keratins and keratin-associated proteins (KRTAPs) are the main components of mammalian nails and hair. Comparative genomics and gene expression studies have revealed that keratins are conserved in all vertebrates, whereas KRTAPs exist only in mammals. Recently, we found hair keratin-like cysteine-rich keratins in jawless vertebrates with confirmed expression in the cornified epithelial teeth of the sea lamprey (*Petromyzon marinus*). Here, we report that KRTAP-like proteins are also present in the horny teeth of the lamprey. Mass spectrometry-based proteomics identified proteins that share features with KRTAPs, such as high contents of cysteine and tyrosine residues, which support intermolecular interactions, and abundant glycine residues, which endow the proteins with flexibility. Genes encoding KRTAP-like proteins are arranged in a cluster in *P. marinus*, and the presence of at least one KRTAP-like protein is conserved in phylogenetically diverse species of lamprey, including *Lampetra fluviatilis*, *Lethenteron reissneri*, *Geotria australis*, and *Mordacia mordax*. The KRTAP-like genes of lampreys contain two exons, whereas mammalian KRTAPs have only a single exon. Although KRTAPs and KRTAP-like proteins are products of independent evolution, their common expression in cornified skin appendages suggests that they fulfill similar functions.

## 1. Introduction

Skin appendages, such as hair, nails, feathers, and scales, are prominent traits of amniotes [1,2,3,4,5]. Hairs are characteristic skin appendages of mammals, and feathers are characteristic for birds. However, cornified skin structures are also found in other vertebrates. Among amphibians, several clades of frogs and salamanders have claws [6] and/or a cornified beak in the developmental stage of tadpoles [7]. Breeding tubercles develop on some teleost fish [8,9], but generally, jawed fish do not have cornified skin appendages. By contrast, all jawless vertebrates (cyclostomes), comprising lampreys and hagfish, form cornified epithelial teeth [10,11,12,13].

**Figure 1:**
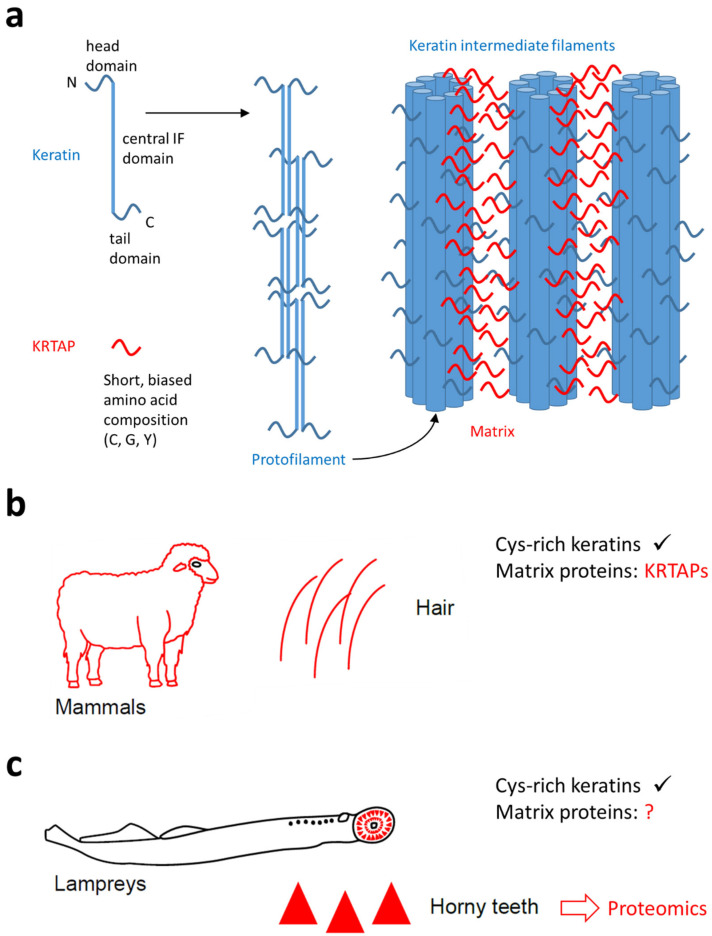
The function of keratin-associated proteins (KRTAPs) in mammalian hair and the possible role of similar proteins in the horny teeth of lampreys. (**a**) A schematic depiction of the contributions of keratins and KRTAPs to the filamentous matrix structure of mammalian hair. (**b**) Mammalian hair contains cysteine (Cys)-rich keratins and KRTAPs, which function as matrix proteins. (**c**) Lampreys have horny teeth in which Cys-rich keratins have also been detected [31]. The contribution of matrix proteins is currently unknown (question mark). The present study uses data from proteomics to screen for matrix proteins of lamprey horny teeth. The symbols in panels (**b**,**c**) are reproduced from a recent open access article [31] which was distributed under the terms of the Creative Commons Attribution License (https://creativecommons.org/licenses/by/4.0/, accessed on 7 March 2025).

Cornified skin appendages consist of dead epithelial cells that are packed with specific proteins [4]. The main protein components of hair and nails are keratins, which form intermediate filaments, and keratin-associated proteins (KRTAPs) [14,15]. The latter are relatively small proteins that share neither a specific fold nor a specific sequence motif, but they fall into clearly defined families known as high-sulfur (HS), ultra-high-sulfur (UHS), and high-glycine–tyrosine (HGT) proteins [16,17,18]. Members of these KRTAP families are either rich in cysteine residues (HS and UHS KRTAPs) or rich in glycine and tyrosine residues (HGT KRTAPs). The common ancestry of KRTAPs is supported by the fact that they all share the same gene structure, that is, a single exon containing the entire coding sequence, and many of the gene family members are arranged in clusters that have apparently evolved by repeated gene duplications [19,20,21]. One cluster of *KRTAP* genes, localized on chromosome 17q21.2, comprises KRTAPs of the HS and UHS families, and another cluster, localized on chromosome 21q22.1, comprises HS KRTAPs and HGT KRTAPs [22]. Given that the *KRTAP* gene cluster on chromosome 17q21.2 is located within the type I keratin gene cluster, it is conceivable that the primordial *KRTAP* gene evolved from a keratin gene [23]. Specifically, *KRTAP*s may be derived from the first exon of a keratin gene because this exon encodes a protein segment which lacks the predominantly alpha-helical structure of the central rod domain and mediates protein–protein interactions in a way similar to KRTAPs [24,25]. Thus, while keratin intermediate filaments provide the core elements of the epithelial cytoskeleton [26,27], the matrix-forming KRTAPs bind them together (Figure 1). KRTAPs are absent from soft epithelia and present in hard mammalian epithelial structures, such as hair, quills, nails, bovine horn, rhino horn, baleen of whales, and filiform papillae on the dorsal tongue surface of many species [28,29,30,31].

Numerous publications have used the term “keratin-associated proteins” to generally refer to proteins that bind to keratins [32]. Examples of such proteins include corneous beta proteins (CBPs), also called beta-keratins, which form both 3.4 nm diameter filaments and the matrix that lies between them [33]. Other examples are proteins encoded by genes of the epidermal differentiation complex both in sauropsids and mammals [4]. Two of them are filaggrin, a keratin filament aggregating protein of the S100 fused-type protein family [34], and trichohyalin, which contains a specific sequence motif implicated in the binding to keratins [35].

Based on protein extraction and two-dimensional electrophoretic analysis, the horny teeth of the Lombardy lamprey (*Lethenteron zanandreai*) were reported to contain both keratins and keratin-associated proteins [12]. Recently, we could show by mass spectrometry-based proteomic analysis that the horny teeth of the sea lamprey (*Petromyzon marinus*) contain cysteine-rich keratins which have evolved independently from mammalian hair keratins and sauropsid-specific hard keratins [31,36]. Here, we searched for KRTAP-like proteins in the proteome of lamprey teeth and corresponding genes in the genomes of jawless vertebrates.

## 2. Materials and Methods

### 2.1. Comparative Genomics

The following genome sequences of jawless vertebrates were investigated: *Petromyzon marinus* (sea lamprey) genome assembly: GCF_010993605.1, assembly name: kPetMar1.pri, assembly provider: Vertebrate Genomes Project, annotation name: Petromyzon marinus annotation release 100, annotation provider: NCBI; *Lethenteron reissneri* (Far Eastern brook lamprey) genome assembly: GCF_015708825.1, assembly name: ASM1570882v1, assembly provider: Center for Ecological and Environmental Sciences [37], annotation name: GCF_015708825.1-RS_2023_12, annotation provider: NCBI RefSeq. Furthermore, we investigated the unannotated whole-genome shotgun sequences of *Mordacia mordax* (Australian lamprey), GenBank accession number JBCLOA000000000.1, and *Geotria australis* (pouched lamprey), GenBank accession number JBCLOB000000000.1 (both submitted by Hardy, C.M.; Court, L.; Rane, R.; Walsh, T.; and Pandey, G., H&B and Environment, CSIRO Applied Genomics Initiative, Parkville, Victoria, Australia), as well as river lamprey (*Lampetra fluviatilis*), GenBank accession number CAXMYT000000000.1, submitted by the Wellcome Sanger Tree of Life Programme, Wellcome Genome Campus, Hinxton, U.K.

*KRTAP-like* genes were first identified in the current genome annotation of the sea lamprey. The gene *LOC116956410* was annotated as “keratin-associated protein 5-1-like [Petromyzon marinus (sea lamprey)]”. Using the encoded protein, XP_032833892.1, as a query in tBLASTn searches, we screened for similar genes adjacent to the locus of *LOC116956410* and in the entire genome of the sea lamprey. Additional BLAST searches were performed on other cyclostome genome sequences available in GenBank. Amino acid sequences were aligned with MUltiple Sequence Comparison by Log-Expectation (MUSCLE) [38] and MultAlin [39].

### 2.2. Analysis of Proteome Data of Sea Lamprey

The proteome of horny teeth of the sea lamprey (Proteomics Identification database, PRIDE, accession number PXD048873) [40] was screened for proteins with sequence features similar to those of mammalian KRTAPs. Cornified teeth and the skin of a sea lamprey specimen (inventory number NMW-63577, kindly provided by the Natural History Museum Vienna) were lysed in a buffer containing urea, thiourea, 3-([3-Cholamidopropyl]dimethylammonio)-2-hydroxy-1-propanesulfonate, and dithiothreitol and analyzed by mass spectrometry-based proteomics, as reported previously [40]. Proteins were identified using the “NCBI_Petromyzon_marinus_tx7757_230919.fasta” dataset, which was downloaded from the Common Repository of Adventitious Proteins (https://www.thegpm.org/crap/, last accessed on 7 March 2025). To test whether the peptide sequences observed are present only in lamprey KRTAPLs or if they could have come from human contaminants, we used the sequences as queries in BLASTp searches in the human proteome. The absence of BLAST hits indicated that the peptides were not derived from contaminating human KRTAPs or other human proteins.

## 3. Results

### 3.1. Prediction of KRTAP-like Genes in Sea Lamprey

**Figure 2:**
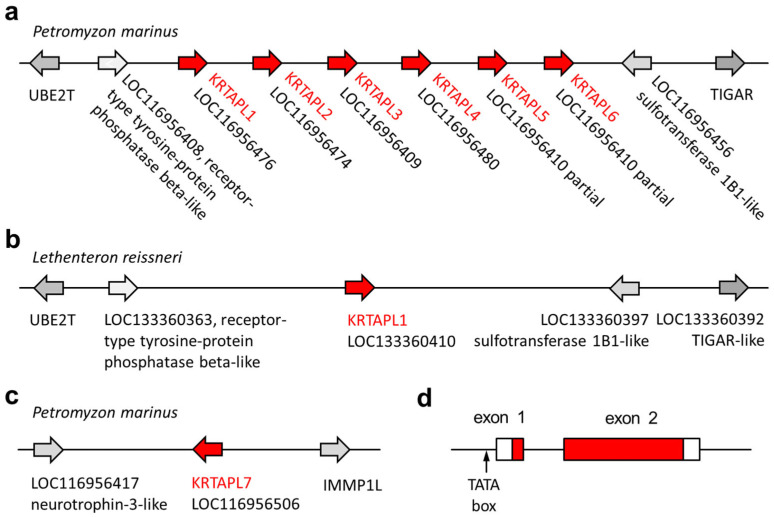
The loci of genes encoding KRTAP-like (KRTAPL) proteins in lampreys. The arrangement of *KRTAPL* genes of the sea lamprey (*Petromyzon marinus*) (**a**,**c**) and the Far Eastern brook lamprey (*Lethenteron reissneri*) (**b**) is schematically depicted. The genes are shown as arrows that point in the direction of transcription. The gene names currently used in GenBank are shown in addition to the annotation as KRTAPLs. (**d**) The organization of *KRTAPL* genes is depicted with boxes representing exons, with red shading indicating protein-coding segments.

We compared lamprey tooth proteins identified by proteomic analysis [31,40] to proteins of other vertebrates and localized the genes that encode these proteins in the genome sequence of the sea lamprey [41,42]. A group of *KRTAP*-like (*KRTAPL*) genes, tentatively named *KRTAPL1* through *KRTAPL6*, was identified in the form of a gene cluster on chromosome 65 of the sea lamprey (Figure 2a). A *KRTAP*-like gene is present at a syntenic locus on chromosome 68 of the Far Eastern brook lamprey (*Lethenteron reissneri*), which is the second species of lampreys for which a chromosome-level genome assembly is available (Figure 2b). At another locus on chromosome 65 of the sea lamprey, a further *KRTAP*-like gene, *KRTAPL7*, was identified (Figure 2c).

The *KRTAP*-like genes of lampreys comprise two exons, which both contain protein-coding sequences (Figure 2d). Important sequence elements, such as a canonical TATA box in the proximal promoter, a canonical splice donor site, and a canonical splice acceptor site are conserved in *KRTAPL* orthologs of different species of lampreys (Figure S1), including the pouched lamprey (*Geotria australis*) and the Australian lamprey (*Mordacia mordax*), which diverged from the lineage leading to the sea lamprey more than 90 million years ago [43]. *KRTAPL3* deviates from the consensus gene organization of *KRTAPL*s because the first in-frame start codon is located in the second exon. *KRTAPL5* and *KRTAPL6* are canonical *KRTAPL*s but have been erroneously combined in a single gene prediction with three exons in the currently available genome annotation of the sea lamprey (Figure S2).

### 3.2. The Detection of KRTAP-like Proteins in the Horny Teeth of the Sea Lamprey

**Figure 3:**
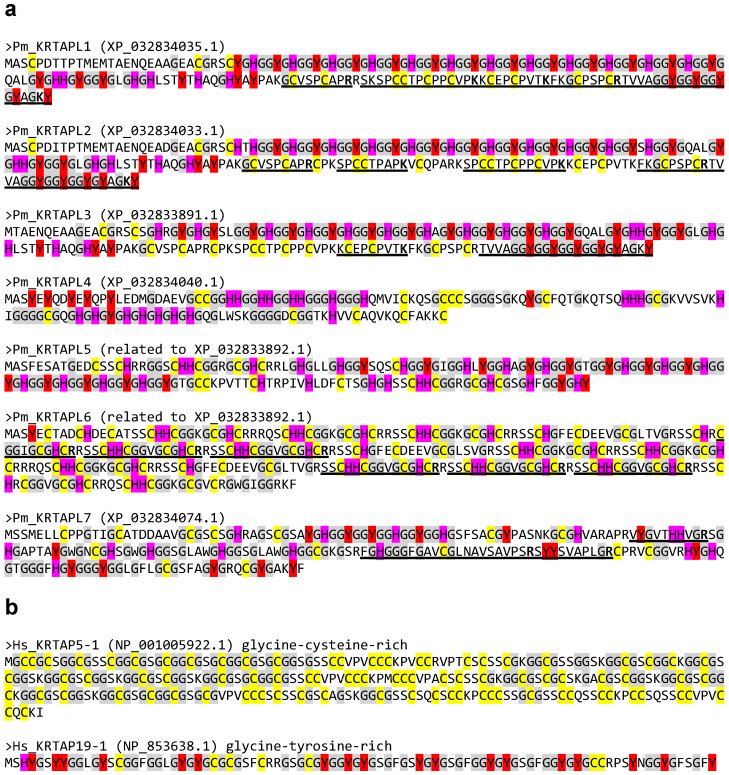
Amino acid sequences of lamprey KRTAP-like proteins (KRTAPLs) and two human KRTAPs. The amino acid sequences of (**a**) KRTAPLs of the sea lamprey (*Petromyzon marinus*, Pm) and (**b**) two representative KRTAPs of humans (*Homo sapiens*, Hs) are shown. GenBank accession numbers are shown in brackets. Cysteine (C) and tyrosine (Y) residues, which are implicated in intermolecular interactions, are highlighted in yellow and red, respectively. Glycine (G) residues, which provide structural flexibility, are shown on a gray background. Histidine (H), abundant in lamprey KRTAPLs but not in human KRTAPs, is highlighted in magenta. Underlines mark sequences of lamprey KRTAPLs that were detected by the proteomic analysis of horny teeth. Carboxy-terminal residues (R, arginine; K, lysine) of tryptic peptides are indicated by bold fonts.

The analysis of the proteomics data of the horny teeth of the sea lamprey [31,40] (Table S1) revealed the presence of tryptic peptides corresponding to KRTAPL1, KRTAPL2, KRTAPL3, KRTAPL6, and KRTAPL7 (Table 1 and Table S2). Among the 107 proteins identified by proteomics, 5 corresponded to KRTAPLs (Table S1). The positions of the peptides within the KRTAPL proteins are indicated in Figure 3.

KRTAPLs of the sea lamprey are in the molecular mass range of 15–32 kilo-Dalton (Table 2), which is smaller than the molecular masses of keratin intermediate filament proteins [31] but within the range of the molecular masses of mammalian KRTAPs [30]. The isoelectric point of all lamprey KRTAPLs is slightly basic (Table 2).

### 3.3. KRTAP-like Proteins of the Lamprey Are Rich in Glycine, Tyrosine, Cysteine, and Histidine

All lamprey KRTAPLs have a glycine content in the range of 23.2–36.4%, which is similar to the glycine content of prototypical mammalian KRTAPs (Table 2). Four out of seven KRTAPLs have a tyrosine content above 9%, and four out of seven KRTAPLs have a cysteine content above 8%, with KRTAPL6 containing more than 21% cysteine residues (Table 2). Cysteine and tyrosine residues are considered critical for the binding of KRTAPs to keratin intermediate filaments and possibly other proteins [30,44,45]. Notably, lamprey KRTAPLs have a markedly higher histidine content than human KRTAPs of all three subclasses (Table 2). A sequence analysis showed that the biased amino acid composition of lamprey KRTAPLs is mainly caused by the presence of the aforementioned residues within short sequence repeats (Figure S3).

**Figure 4:**
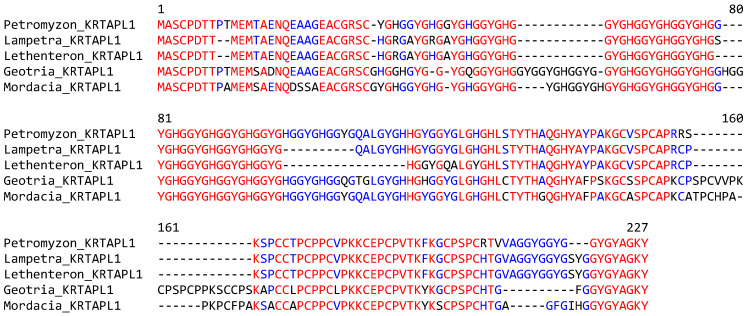
KRTAP-like 1 (KRTAPL1) is conserved in phylogenetically diverse clades of lampreys. The amino acid sequences of KRTAPL1 of five species of lampreys were aligned. Red and blue text indicate the conservation of residues in all and more than half of the species, respectively. Species: sea lamprey (*Petromyzon marinus*), river lamprey (*Lampetra fluviatilis*), Far Eastern brook lamprey (*Lethenteron reissneri*), pouched lamprey (*Geotria australis*), and Australian lamprey (*Mordacia mordax*). The GenBank accession numbers of *KRTAPL1* genes of *Petromyzon marinus*, *Geotria australis* and *Mordacia mordax* are provided in Figure S1. *KRTAPL1* of *Lampetra fluviatilis*: CAXMYT010003053.1, nucleotides 14,580–14,587 (exon 1), and 13,934–13,403 (exon 2). *KRTAPL1* of *Lethenteron reissneri* is available in GenBank under the gene name *LOC133360410*.

The sequence alignment of KRTAPL1 proteins of phylogenetically diverse species of lampreys showed a high degree of conservation over the entire length of the protein, with variation arising mainly from different lengths of repetitive sequences (Figure 4). No orthologs of lamprey *KRTAPL*s were found in whole-genome tBLASTn searches and the detailed analysis of syntenic chromosomal loci (Figure 2) in jawed fishes and tetrapods.

## 4. Discussion

**Figure 5:**
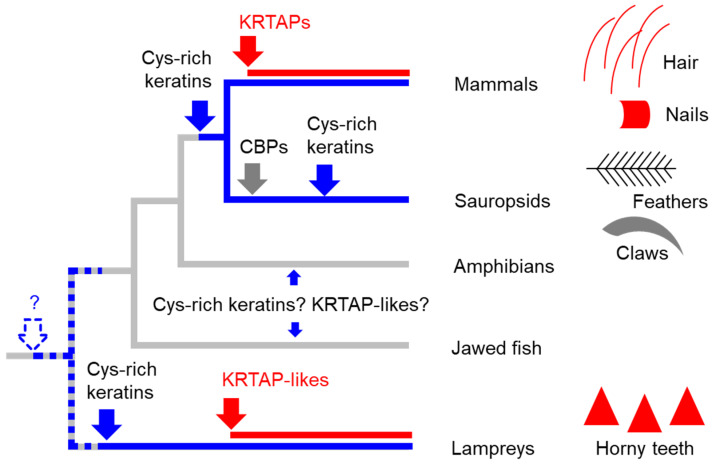
A scenario for the evolution of keratins, KRTAPs, and KRTAP-like proteins in vertebrates. Skin appendages of different clades of vertebrates are schematically depicted, with red symbols indicating the presence of KRTAP or KRTAPL proteins. On the phylogenetic tree, blue and red lines indicate the presence of cysteine (Cys)-rich keratins and KRTAP/KRTAPL-proteins, respectively. Blue, red and gray arrows indicate the origin of Cys-rich keratins, KRTAP/KRTAPL-proteins, and corneous beta-proteins (CBPs) respectively. The dashed arrow and the question mark indicate a possible early origin of Cys-rich keratins in a common ancestor of vertebrates.

The results of this study show a family of proteins that are components of horny teeth. These proteins are relatively small and have an amino acid composition biased towards glycine, tyrosine, and cysteine. In this regard, they are similar to mammalian KRTAPs, which are components of hair, nails, quills, and other hard cornified structures [30,46,47]. KRTAPLs and KRTAPs differ with regard to the gene structure (two exons in KRTAPLs and only one exon in KRTAPs) and the abundance of specific amino acids. For example, arginine is abundant in lamprey KRTAPL6 but not in human KRTAPs, and the histidine content is generally higher in KRTAPLs than in KRTAPs (Table 2). The differences in the gene structure and the species distributions of the genes suggest a model for the evolution of KRTAPLs and KRTAPs, which is depicted in Figure 5. According to this model, the evolution of the horny teeth of lampreys did not only involve the evolution of cysteine-rich keratins [31] but also the evolution of KRTAPLs. The latter are the products of convergent evolution relative to mammalian KRTAPs.

The present study supports and extends the concept that proteins with sequence similarities to mammalian KRTAPs exist in non-mammalian species [48]. A recent study explored the phylogenetic history of KRTAPs and proteins of similar sequence features, but no support for common ancestry was found [49]. The comparison of gene structures and the limitation of sequence similarities to stretches of low sequence complexity indicates that KRTAPs and lamprey KRTAPLs have not evolved from a common ancestral gene. Accordingly, we conclude that non-mammalian taxa including sauropsids (reptiles and birds) and lampreys do not have homologs, i.e., proteins of common evolutionary ancestry of KRTAPs. Given the apparent co-localization of KRTAPs and KRTAPLs with keratins in hard cornified epithelial structures, we propose that the similarities of KRTAPs and KRTAPLs have not arisen by chance but in the course of convergent evolution. The assumptions underlying this hypothesis need to be investigated further.

This study provides new insights into the protein composition of horny teeth. Using proteomics [40], KRTAPLs were detected in the teeth but not in the skin. This pattern fits with the predominant expression of mammalian KRTAPs at sites of hard cornification [30]. However, the absence of detection in the skin cannot be regarded as proof of absence. Therefore, it will be important to determine the tissue expression pattern of *KRTAPL*s in lampreys using alternative methods, such as RNA-seq, RT-PCR, and antibody-dependent methods, such as immunoblot analysis and immunohistochemistry. It will be interesting to study *KRTAPL* gene expression during the development, homeostasis, and regeneration of the horny teeth of lampreys.

The development and differentiation of the horny teeth of lampreys has not been fully characterized so far. Some studies addressed specific questions, such as the roles of sulfhydryl oxidase [50] and transglutaminases [40] in protein cross-linking. Cysteine-rich keratins and KRTAPLs of horny teeth are candidate markers of epithelial differentiation and likely functional equivalents of structural proteins in hard skin appendages of land-dwelling vertebrates [4,51].

Both KRTAPs and KRTAPLs are present in hard cornified epithelial structures and their amino acid compositions are similar. However, it remains to be experimentally tested whether KRTAPs and KRTAPLs have similar functions as matrix proteins between intermediate filaments, as depicted in Figure 1. Investigating the functions of these proteins is difficult because hard cornification cannot be fully mimicked in vitro [52]. Gene knockout studies are presumably complicated by the functional redundancy of genes of the same family, similar to the study of subgroups of keratins [53,54]. Despite these challenges being anticipated, the present study provides a basis for the design of future studies into the molecular structure of cornified teeth in jawless vertebrates.

## Figures and Tables

**Table 1:** KRTAP-like proteins identified by mass spectrometry-based proteomics in horny teeth of *P. marinus*.

Protein	Peptide Sequence	Unique	Percolator q-Value (by Search Engine): Sequest HT	Percolator PEP (by Search Engine): Sequest HT
KRTAPL1	SPCCTPCPPCVPK	yes	0.0003848	3.31 × 10^−7^
	TVVAGGYGGYGGYGYAGK	no	0.0003848	7.20 × 10^−9^
	TVVAGGYGGYGGYGYAGKY	no	0.0003848	5.45 × 10^−8^
	GCVSPCAPR	no	0.0003848	5.29 × 10^−4^
	KCEPCPVTK	no	0.0003848	1.26 × 10^−3^
	SKSPCCTPCPPCVPK	yes	0.001314	5.41 × 10^−3^
	FKGCPSPCR	no	0.001496	6.00 × 10^−3^
KRTAPL2	SPCCTPAPK	yes	0.0003848	1.92 × 10^−4^
	SPCCTPCPPCVPK	no	0.0003848	3.31 × 10^−7^
	TVVAGGYGGYGGYGYAGK	no	0.0003848	7.20 × 10^−9^
	TVVAGGYGGYGGYGYAGKY	no	0.0003848	5.45 × 10^−8^
	GCVSPCAPR	no	0.0003848	5.29 × 10^−4^
	KCEPCPVTK	no	0.0003848	1.26 × 10^−3^
	FKGCPSPCR	no	0.001496	6.00 × 10^−3^
KRTAPL3	SPCCTPCPPCVPK	no	0.0003848	3.31 × 10^−7^
	TVVAGGYGGYGGYGGYGYAGKY	yes	0.0003848	3.59 × 10^−10^
	GCVSPCAPR	no	0.0003848	5.29 × 10^−4^
	KCEPCPVTK	no	0.0003848	1.26 × 10^−3^
	FKGCPSPCR	no	0.001496	6.00 × 10^−3^
KRTAPL6	SSCHHCGGVGCGHCR	yes	0.0003848	3.85 × 10^−6^
	CGGIGCGHCR	yes	0.0003848	1.26 × 10^−6^
KRTAPL7	VYGVTHHVGR	yes	0.0003848	7.14 × 10^−6^
	FGHGGGFGAVCGLNAVSAVPSR *	yes	0.0003848	4.00 × 10^−17^
	FGHGGGFGAVCGLNAVSAVPSR *	yes	0.0003848	3.27 × 10^−17^
	FGHGGGFGAVCGLNAVSAVPSRSYYSVAPLGR	yes	0.0003848	6.14 × 10^−5^
	SYYSVAPLGR	yes	0.0007115	3.06 × 10^−3^

Note: Further data characterizing the detection of peptides are provided in Table S2. *, different modifications (Table S2).

**Table 2:** Molecular weight, isoelectric point, and amino acid composition of lamprey KRTAPLs and human KRTAPs.

Protein:		KRTAPL1	KRTAPL2	KRTAPL3	KRTAPL4	KRTAPL5	KRTAPL6	KRTAPL7	KRTAP5-1	KRTAP19-1
Species:		Lamprey	Lamprey	Lamprey	Lamprey	Lamprey	Lamprey	Lamprey	Human	Human
aa residues:		188	199	173	147	165	310	220	278	90
Mw (kD):		18,942	20,199	17,425	14,964	16,476	32,323	21,436	24,194	9008
pI:		8.50	8.51	8.73	7.98	8.36	8.78	9.03	8.39	8.45
aa composition:										
Ala	A	6.4%	6.5%	6.9%	2.7%	1.8%	1.0%	**8.6%**	1.1%	0.0%
Arg	R	2.1%	2.0%	2.3%	0.0%	4.2%	**11.3%**	4.5%	0.4%	3.3%
Asn	N	0.5%	0.5%	0.6%	0.0%	0.0%	0.0%	1.4%	0.0%	1.1%
Asp	D	0.5%	1.0%	0.0%	2.7%	1.2%	1.6%	0.9%	0.0%	0.0%
Cys	C	6.9%	**8.5%**	7.5%	**8.8%**	**9.1%**	**21.9%**	6.4%	**30.6%**	7.8%
Gln	Q	1.6%	2.0%	1.7%	**8.2%**	0.6%	1.0%	0.9%	1.4%	0.0%
Glu	E	2.7%	2.5%	2.3%	2.7%	1.2%	3.5%	0.5%	0.0%	0.0%
Gly	G	**30.9%**	**26.6%**	**30.6%**	**29.9%**	**36.4%**	**23.2%**	**31.8%**	**31.7%**	**42.2%**
His	H	**9.6%**	**9.0%**	**9.2%**	**13.6%**	**15.8%**	**13.9%**	7.3%	0.0%	1.1%
Ile	I	0.0%	0.5%	0.0%	1.4%	1.2%	0.6%	0.5%	0.4%	0.0%
Leu	L	1.6%	1.5%	2.3%	1.4%	3.0%	1.0%	3.6%	0.0%	2.2%
Lys	K	3.7%	4.5%	4.0%	6.8%	0.6%	2.6%	1.4%	6.1%	0.0%
Met	M	1.6%	1.5%	0.6%	2.0%	0.6%	0.3%	0.9%	0.7%	1.1%
Phe	F	0.5%	0.5%	0.6%	1.4%	1.8%	1.3%	3.2%	0.0%	7.8%
Pro	P	7.4%	**9.5%**	7.5%	0.7%	1.2%	0.0%	3.6%	3.6%	1.1%
Ser	S	3.7%	4.0%	4.0%	4.8%	6.7%	**10.6%**	**8.6%**	**19.8%**	**12.2%**
Thr	T	4.8%	5.0%	3.5%	2.0%	4.2%	1.3%	2.3%	0.4%	0.0%
Trp	W	0.0%	0.0%	0.0%	0.7%	0.0%	0.3%	1.8%	0.0%	0.0%
Tyr	Y	**12.8%**	**11.1%**	**13.3%**	4.8%	**9.1%**	0.3%	6.8%	0.0%	**20.0%**
Val	V	2.7%	3.0%	2.9%	5.4%	1.2%	4.2%	5.0%	4.0%	0.0%

Notes: aa, amino acid; kD, kilodalton; Mw, molecular weight; pI, isoelectric point; KRTAP, keratin-associated protein; KRTAPL, KRTAP-like. Lamprey: *Petromyzon marinus*. Amino acids are shown in both 3-letter and 1-letter codes. Percentages larger than 8% are shown in bold text.

## Data Availability

All data generated and analyzed in this study are included in this article.

## Supplementary Materials

The following supporting information can be downloaded at https://www.mdpi.com/article/10.3390/jdb13020018/s1, Figure S1: Conservation of *KRTAPL1* gene structure and nucleotide sequence in three species of lampreys; Figure S2: Prediction of *KRTAPL5* and *KRTAPL6* genes in comparison to *LOC116956410*; Figure S3: Lamprey KRTAP-like proteins contain sequence repeats; Table S1: Mass spectrometry-based proteomics of horny teeth of sea lamprey identifying KRTAP-like (KRTAPL) proteins; Table S2: KRTAP-like peptides identified by mass spectrometry-based proteomics.
