# Supplementary material for: Cornified Epithelial Teeth of Jawless Vertebrates Contain Proteins Similar to Keratin-Associated Proteins of Mammalian Skin Appendages
Source: J Dev Biol. 2025 May 19;13(2):18. doi: 10.3390/jdb13020018 (PMC12101200; doi:10.3390/jdb13020018)
Supplement: Supplementary file 1 [file jdb-13-00018-s001.zip › jdb-3545005-supplementary.pdf]

Table S1. Mass spectrometry-based proteomics of horny teeth of the sea lamprey identifies KRTAP-like (KRTAPL) proteins

Rank	Accession (GenBank)	Description (NCBI Petromyzon marinus Annotation Release 100)	Protein (this study)	# AAs	Coverage [%]	# Peptides	# Unique Peptides	# Peptide spectrum matches	Score Sequest HT: Sequest HT
1	XP_032832261.1	keratin, type I cytoskeletal 19-like [Petromyzon marinus]	KRT	458	99	66	66	984	1974,87
2	XP_032836208.1	keratin, type II cytoskeletal cochleal-like [Petromyzon marinus]	KRT	521	74	65	61	927	1798,83
3	XP_032812944.1	keratin, type II cytoskeletal 8-like [Petromyzon marinus]	KRT	495	81	70	34	431	867,38
4	XP_032814531.1	keratin, type 1 cytoskeletal 11-like [Petromyzon marinus]	KRT	448	71	57	48	430	818,59
5	XP_032812945.1	keratin, type II cytoskeletal 8-like [Petromyzon marinus]	KRT	484	76	55	12	233	383,06
6	XP_032812943.1	keratin, type II cytoskeletal 8-like [Petromyzon marinus]	KRT	510	75	50	22	210	372,49
7	XP_032834074.1	glycine-rich cell wall structural protein 2-like [Petromyzon marinus]	KRTAPL7	220	19	4	4	98	184,01
8	XP_032808690.1	microtubule-actin cross-linking factor 1-like [Petromyzon marinus]	-	2636	27	59	59	113	167,65
9	XP_032820218.1	cadherin-2-like [Petromyzon marinus]	-	1008	21	15	15	46	101,57
10	XP_032816031.1	plakophilin-3-like [Petromyzon marinus]	-	942	33	28	28	48	96,21
11	XP_032806953.1	catenin beta-1-like [Petromyzon marinus]	-	780	25	16	14	43	80,97
12	XP_032817362.1	fatty acid-binding protein, heart-like [Petromyzon marinus]	-	133	50	8	8	38	63,63
13	XP_032809983.1	filamin-A-like [Petromyzon marinus]	-	2528	11	18	18	30	62,92
14	XP_032817193.1	arachidonate 5-lipoxygenase-like [Petromyzon marinus]	-	668	31	18	18	41	62,64
15	XP_032837300.1	keratin, type 1 cytoskeletal 11-like [Petromyzon marinus]	KRT	387	18	9	1	43	61,58
16	XP_032800492.1	keratin, type 1 cytoskeletal 11-like, partial [Petromyzon marinus]	KRT	409	20	12	2	44	61,01
17	AJG41731.1	beta-actin [Petromyzon marinus]	-	376	49	13	3	30	50,19
18	XP_032816216.1	protein-glutamine gamma-glutamyltransferase 4-like [Petromyzon marinus]	-	787	31	18	18	30	45,50
19	XP_032816807.1	uncharacterized protein LOC116946080 [Petromyzon marinus]	-	217	46	9	9	22	43,45
20	XP_032801611.1	intermediate filament protein ON3-like [Petromyzon marinus]	-	476	13	7	1	38	43,26
21	XP_032817105.1	actin, cytoplasmic 2-like [Petromyzon marinus]	-	376	31	10	1	23	40,78
22	XP_032804442.1	actin, alpha cardiac muscle 2 [Petromyzon marinus]	-	377	24	8	2	18	32,67
23	XP_032801610.1	keratin, type II cytoskeletal 8-like [Petromyzon marinus]	KRT	475	6	3	1	23	25,48
24	XP_032802783.1	glyceraldehyde-3-phosphate dehydrogenase [Petromyzon marinus]	-	333	12	3	3	10	25,37
25	XP_032832439.1	tubulin beta-4 chain-like [Petromyzon marinus]	-	445	16	6	6	16	23,40
26	XP_032822326.1	uncharacterized protein LOC116949299 isoform X1 [Petromyzon marinus]	-	920	7	4	2	12	23,06
27	XP_032834033.1	abscisic acid and environmental stress-inducible protein-like [Petromyzon marinus]	KRTAPL2	199	34	7	1	12	22,88
28	XP_032823532.1	clustered mitochondria protein homolog isoform X1 [Petromyzon marinus]	-	2053	5	8	8	11	22,42
29	XP_032806848.1	lamin-B1-like isoform X1 [Petromyzon marinus]	-	783	17	11	11	15	21,87
30	XP_032803858.1	annexin A2-like [Petromyzon marinus]	-	332	14	5	5	8	21,03
31	XP_032834035.1	cold and drought-regulated protein CORA-like [Petromyzon marinus]	KRTAPL1	188	32	7	1	12	20,74
32	XP_032824491.1	elongation factor 2 [Petromyzon marinus]	-	858	8	4	4	8	19,39
33	XP_032825520.1	protein-arginine deiminase type-2-like [Petromyzon marinus]	-	992	10	9	9	12	19,10
34	XP_032803022.1	14-3-3 protein gamma-A-like [Petromyzon marinus]	-	248	19	5	4	9	18,70
35	XP_032800944.1	serum albumin SDS-1 [Petromyzon marinus]	-	1423	8	9	9	10	18,13
36	XP_032834085.1	filaggrin-2-like [Petromyzon marinus]	-	1141	5	5	5	9	17,52
37	XP_032823604.1	14-3-3 protein epsilon-like [Petromyzon marinus]	-	258	16	4	3	8	17,18
38	XP_032819215.1	tubulin alpha chain, testis-specific-like [Petromyzon marinus]	-	450	13	4	2	10	15,59
39	XP_032833891.1	cold and drought-regulated protein CORA-like [Petromyzon marinus]	KRTAPL3	173	36	5	1	8	14,64
40	XP_032815474.1	catenin beta-1 [Petromyzon marinus]	-	777	5	3	1	8	14,60
41	AAN73382.1	ribosomal protein L18, partial [Petromyzon marinus]	-	188	23	4	4	6	13,81
42	XP_032811538.1	40S ribosomal protein S3 [Petromyzon marinus]	-	249	16	4	4	5	13,79
43	XP_032819572.1	histone H4 [Petromyzon marinus]	-	103	51	6	6	8	13,26
44	XP_032829724.1	pyruvate kinase PKM [Petromyzon marinus]	-	529	12	5	5	6	12,93
45	XP_032811593.1	calpain-1 catalytic subunit-like [Petromyzon marinus]	-	878	8	6	6	7	12,78
46	XP_032820277.1	heat shock cognate 71 kDa protein isoform X1 [Petromyzon marinus]	-	651	7	5	5	6	12,53
47	AAA49267.1	lactate dehydrogenase [Petromyzon marinus]	-	334	15	4	4	5	11,84
48	XP_032833791.1	profilin-1-like [Petromyzon marinus]	-	139	27	2	2	6	11,82
49	XP_032817266.1	LOW QUALITY PROTEIN: plectin-like [Petromyzon marinus]	-	2752	12	7	2	7	11,02
50	XP_032811511.1	plectin-like [Petromyzon marinus]	-	897	5	6	6	6	10,74
51	XP_032803290.1	annexin A2 isoform X1 [Petromyzon marinus]	-	396	9	3	3	4	10,23
52	XP_032833379.1	hydroxyacylglutathione hydrolase, mitochondrial isoform X1 [Petromyzon marinus]	-	324	8	2	2	3	9,90
53	XP_032817272.1	LOW QUALITY PROTEIN: plectin-like [Petromyzon marinus]	-	2038	11	7	2	7	9,81
54	P07096.1	RecName: Full=Blood plasma apolipoprotein LAL2; Flags: Precursor	-	191	34	5	5	8	9,67
55	XP_032801167.1	nucleoside diphosphate kinase-like isoform X1 [Petromyzon marinus]	-	149	22	4	4	4	9,63
56	XP_032809885.1	transitional endoplasmic reticulum ATPase-like [Petromyzon marinus]	-	811	4	3	3	4	8,46
57	XP_032799905.1	T-complex protein 1 subunit zeta [Petromyzon marinus]	-	531	6	3	3	5	8,12
58	XP_032805864.1	retinal dehydrogenase 2-like [Petromyzon marinus]	-	520	8	3	3	4	7,53
59	XP_032800408.1	T-complex protein 1 subunit alpha isoform X3 [Petromyzon marinus]	-	559	9	5	5	6	7,39
60	XP_032830674.1	40S ribosomal protein SA [Petromyzon marinus]	-	298	13	3	3	4	7,11
61	XP_032824816.1	ATP synthase subunit alpha, mitochondrial isoform X1 [Petromyzon marinus]	-	553	7	3	3	4	6,87
62	XP_032831352.1	ATP synthase subunit beta, mitochondrial [Petromyzon marinus]	-	524	8	3	3	4	6,68
63	XP_032815375.1	apolipoprotein B-100 [Petromyzon marinus]	-	5361	1	4	4	4	6,65
64	XP_032806935.1	myosin light polypeptide 6-like isoform X1 [Petromyzon marinus]	-	151	13	2	2	4	6,53
65	XP_032817138.1	myosin-10-like isoform X1 [Petromyzon marinus]	-	1981	1	3	3	4	6,33
66	XP_032807738.1	vimentin-like [Petromyzon marinus]	-	446	4	2	2	11	6,30
67	XP_032816217.1	protein-glutamine gamma-glutamyltransferase K-like [Petromyzon marinus]	-	811	3	3	3	3	6,17
68	XP_032812044.1	alpha-actinin-1-like isoform X1 [Petromyzon marinus]	-	890	6	4	4	4	5,90
69	XP_032828431.1	40S ribosomal protein S4, X isoform [Petromyzon marinus]	-	263	11	3	3	4	5,84
70	XP_032813838.1	T-complex protein 1 subunit beta [Petromyzon marinus]	-	535	5	2	2	2	5,59
71	XP_032817596.1	elongation factor 1-alpha, somatic form [Petromyzon marinus]	-	463	8	2	2	3	5,52
72	XP_032815154.1	CD109 antigen-like [Petromyzon marinus]	-	1410	4	4	4	4	5,50
73	AAZ83742.1	carbonic anhydrase [Petromyzon marinus]	-	262	9	3	3	3	5,48
74	XP_032819329.1	filamin-A-like isoform X1 [Petromyzon marinus]	-	2608	2	3	3	4	5,34
75	XP_032819899.1	cytoplasmic dynein 1 heavy chain 1-like isoform X1 [Petromyzon marinus]	-	4637	1	3	3	4	5,33
76	XP_032833892.1	keratin-associated protein 5-1-like [Petromyzon marinus]	KRTAPL5/6	468	18	2	2	5	5,31
77	XP_032820625.1	calpain-2 catalytic subunit-like [Petromyzon marinus]	-	944	3	2	2	2	5,12
78	XP_032831572.1	pyruvate kinase PKM-like isoform X1 [Petromyzon marinus]	-	532	4	2	2	2	5,11
79	XP_032818217.1	enolase [Petromyzon marinus]	-	434	7	2	2	3	5,01
80	XP_032804478.1	calpain-1 catalytic subunit-like [Petromyzon marinus]	-	706	4	3	3	3	4,95
81	XP_032810903.1	plectin-like [Petromyzon marinus]	-	745	8	3	3	3	4,90
82	AAM88904.1	guanine nucleotide-binding protein [Petromyzon marinus]	-	317	7	2	2	3	4,54
83	XP_032806574.1	eukaryotic translation initiation factor 3 subunit A isoform X1 [Petromyzon marinus]	-	1373	3	3	3	3	4,46
84	XP_032818000.1	proteasome subunit beta type-6 [Petromyzon marinus]	-	233	5	2	2	2	4,45
85	XP_032834739.1	prosaposin-like [Petromyzon marinus]	-	372	7	2	2	2	4,32
86	XP_032834804.1	protein-glutamine gamma-glutamyltransferase K-like isoform X1 [Petromyzon marinu	-	856	3	2	2	2	4,30
87	XP_032801469.1	alkaline phosphatase, tissue-nonspecific isozyme, partial [Petromyzon marinus]	-	347	8	2	2	2	4,25
88	XP_032814482.1	40S ribosomal protein S26 [Petromyzon marinus]	-	118	16	2	2	2	4,12
89	XP_032831750.1	T-complex protein 1 subunit gamma [Petromyzon marinus]	-	560	5	2	1	3	4,01
90	XP_032836002.1	eIF-2-alpha kinase activator GCN1 [Petromyzon marinus]	-	2694	2	3	3	3	3,97
91	XP_032830511.1	gigaxonin [Petromyzon marinus]	-	618	4	2	2	2	3,96
92	XP_032837319.1	keratin, type I cytoskeletal 13-like [Petromyzon marinus]	KRT	829	3	2	2	2	3,84
93	XP_032826392.1	cuticle collagen 6-like [Petromyzon marinus]	-	191	12	2	2	2	3,75
94	XP_032834723.1	aerolysin-like protein [Petromyzon marinus]	-	313	10	2	2	2	3,64
95	XP_032813046.1	uncharacterized protein LOC116943873 [Petromyzon marinus]	-	1706	1	2	2	2	3,57
96	XP_032834312.1	transmembrane protein 47-like [Petromyzon marinus]	-	180	11	2	2	2	3,51
97	XP_032815156.1	CD109 antigen-like isoform X1 [Petromyzon marinus]	-	1443	2	2	2	2	3,03
98	XP_032832495.1	cystatin-B-like [Petromyzon marinus]	-	99	19	2	2	2	2,20
99	XP_032814865.1	glutathione peroxidase 6-like [Petromyzon marinus]	-	145	8	2	2	2	2,20
100	XP_032833350.1	LOW QUALITY PROTEIN: coagulation factor XIII A chain-like [Petromyzon marinus]	-	804	3	2	2	2	2,18
101	XP_032831821.1	perilipin-3-like [Petromyzon marinus]	-	402	4	2	2	2	2,05
102	XP_032822285.1	actin-related protein 2/3 complex subunit 4 [Petromyzon marinus]	-	168	11	2	2	2	1,95
103	XP_032803479.1	copper chaperone for superoxide dismutase [Petromyzon marinus]	-	279	14	3	3	3	1,89
104	XP_032802310.1	aldo-keto reductase family 1 member B7-like [Petromyzon marinus]	-	335	5	2	2	2	1,75
105	XP_032817391.1	myosin regulatory light chain 12A [Petromyzon marinus]	-	174	23	2	2	3	1,65
106	XP_032829378.1	annexin A4-like [Petromyzon marinus]	-	524	3	2	2	2	0,00
107	XP_032830825.1	T-complex protein 1 subunit eta [Petromyzon marinus]	-	539	4	2	1	2	0,00

Notes: AAs, amino acids; KRT, keratin; KRTAPL, keratin-associated protein-like. Protein (this study): only KRTs and KRTAPLs are highlighted.

The peptides of keratin-associated protein 5-1-like [Petromyzon marinus], accession number XP_032833892.1 (KRTAPL5/6) correspond to the prediction of KRTAPL6 of this study (see Figure S2).

The primary data of this table were published by Sachslehner et al. Mol Biol Evol. 2024;41:msae100. doi: 10.1093/molbev/msae100.

Table S2. KRTAP-like peptides identified by mass spectrometry-based proteomics

Protein	Peptide sequence	Modifications	Qvality PEP	Qvality q-value	# Protein Groups	# Proteins	# PSMs	Master Protein Accessions	# Missed Cleavages	Theo. MH+ [Da]	Confidence (by Search Engine): Sequest HT	Percolator q-Value (by Search Engine): Sequest HT	Percolator PEP (by Search Engine): Sequest HT	XCorr (by Search Engine): Sequest HT
KRTAPL1	SPCCTPCPPCVPK	4xCarbamidomethyl [C3; C4; C7; C10]	1,53E-05	0,000820	3	3	2	XP_032834035.1; XP_032834033.1; XP_032833891.1	0	1559,64734	High	0,0003848	3,306E-07	2,4
	TVVAGGYGGYGGYGYAGK		7,71E-07	0,000820	2	2	1	XP_032834035.1; XP_032834033.1	0	1696,79656	High	0,0003848	7,203E-09	2,91
	TVVAGGYGGYGGYGYAGKY		3,75E-06	0,000820	2	2	3	XP_032834035.1; XP_032834033.1	1	1859,85989	High	0,0003848	5,454E-08	3,09
	GCVSPCAPR	2xCarbamidomethyl [C2; C6]	4,87E-03	0,000820	3	3	3	XP_032834035.1; XP_032834033.1; XP_032833891.1	0	1003,4448	High	0,0003848	0,0005293	2,01
	KCEPCPVTK	2xCarbamidomethyl [C2; C5]	9,65E-03	0,000820	3	3	1	XP_032834035.1; XP_032834033.1; XP_032833891.1	1	1118,53328	High	0,0003848	0,001262	2,09
	SKSPCCTPCPPCVPK	4xCarbamidomethyl [C5; C6; C9; C12]	3,12E-02	0,003100	1	1	1	XP_032834035.1	1	1774,77433	High	0,001314	0,005409	1,23
	FKGCPSPCR	2xCarbamidomethyl [C4; C8]	3,41E-02	0,003581	3	3	1	XP_032834035.1; XP_032834033.1; XP_032833891.1	1	1108,50265	High	0,001496	0,006002	1,89
KRTAPL2	SPCCTPAPK	2xCarbamidomethyl [C3; C4]	2,19E-03	0,000820	1	1	1	XP_032834033.1	0	1017,44921	High	0,0003848	0,0001915	2,15
	SPCCTPCPPCVPK	4xCarbamidomethyl [C3; C4; C7; C10]	1,53E-05	0,000820	3	3	2	XP_032834035.1; XP_032834033.1; XP_032833891.1	0	1559,64734	High	0,0003848	3,306E-07	2,4
	TVVAGGYGGYGGYGYAGK		7,71E-07	0,000820	2	2	1	XP_032834035.1; XP_032834033.1	0	1696,79656	High	0,0003848	7,203E-09	2,91
	TVVAGGYGGYGGYGYAGKY		3,75E-06	0,000820	2	2	3	XP_032834035.1; XP_032834033.1	1	1859,85989	High	0,0003848	5,454E-08	3,09
	GCVSPCAPR	2xCarbamidomethyl [C2; C6]	4,87E-03	0,000820	3	3	3	XP_032834035.1; XP_032834033.1; XP_032833891.1	0	1003,4448	High	0,0003848	0,0005293	2,01
	KCEPCPVTK	2xCarbamidomethyl [C2; C5]	9,65E-03	0,000820	3	3	1	XP_032834035.1; XP_032834033.1; XP_032833891.1	1	1118,53328	High	0,0003848	0,001262	2,09
	FKGCPSPCR	2xCarbamidomethyl [C4; C8]	3,41E-02	0,003581	3	3	1	XP_032834035.1; XP_032834033.1; XP_032833891.1	1	1108,50265	High	0,001496	0,006002	1,89
KRTAPL3	SPCCTPCPPCVPK	4xCarbamidomethyl [C3; C4; C7; C10]	1,53E-05	0,000820	3	3	2	XP_032834035.1; XP_032834033.1; XP_032833891.1	0	1559,64734	High	0,0003848	3,306E-07	2,4
	TVVAGGYGGYGGYGGYGYAGKY		7,44E-08	0,000820	1	1	1	XP_032833891.1	1	2136,96615	High	0,0003848	3,592E-10	4,24
	GCVSPCAPR	2xCarbamidomethyl [C2; C6]	4,87E-03	0,000820	3	3	3	XP_032834035.1; XP_032834033.1; XP_032833891.1	0	1003,4448	High	0,0003848	0,0005293	2,01
	KCEPCPVTK	2xCarbamidomethyl [C2; C5]	9,65E-03	0,000820	3	3	1	XP_032834035.1; XP_032834033.1; XP_032833891.1	1	1118,53328	High	0,0003848	0,001262	2,09
	FKGCPSPCR	2xCarbamidomethyl [C4; C8]	3,41E-02	0,003581	3	3	1	XP_032834035.1; XP_032834033.1; XP_032833891.1	1	1108,50265	High	0,001496	0,006002	1,89
KRTAPL5/6	SSCHHCGGVGCGHCR	4xCarbamidomethyl [C3; C6; C11; C14]	1,04E-04	0,000820	1	1	3	XP_032833892.1	0	1727,63661	High	0,0003848	0,000003849	1,84
	CGGIGCGHCR	3xCarbamidomethyl [C1; C6; C9]	4,35E-05	0,000820	1	1	2	XP_032833892.1	0	1133,43973	High	0,0003848	0,000001258	2,75
KRTAPL7	VYGVTHHVGR		1,68E-04	0,000820	1	1	11	XP_032834074.1	0	1124,59595	High	0,0003848	0,00000714	2,13
	FGHGGGFGAVCGLNAVSAVPSR	1xCarbamidomethyl [C11]	2,78E-13	0,000820	1	1	36	XP_032834074.1	0	2117,03452	High	0,0003848	4,001E-17	4,74
	FGHGGGFGAVCGLNAVSAVPSR	1xCarbamidomethyl [C11]; 1xDeamidated [N14]	2,37E-13	0,000820	1	1	22	XP_032834074.1	0	2118,01853	High	0,0003848	3,265E-17	5,45
	FGHGGGFGAVCGLNAVSAVPSRSYYSVAPLGR	1xCarbamidomethyl [C11]; 1xDeamidated [N14]	9,03E-04	0,000820	1	1	2	XP_032834074.1	1	3211,57418	High	0,0003848	0,00006135	2,28
	SYYSVAPLGR		1,96E-02	0,001621	1	1	27	XP_032834074.1	0	1112,57348	High	0,0007115	0,003057	2,12

Notes: PSMs, peptide spectrum matches; Xcorr, cross correlation score (Eng JK, Fischer B, Grossmann J, Maccoss MJ. A fast SEQUEST cross correlation algorithm. J Proteome Res. 2008;7:4598-4602. doi: 10.1021/pr800420s).

```
            1                                                                                                  100
Petromyzon  TATAAAGGGGCCCATAAAAGCAGAGAGAAGTCACAGCGATCTGTCAGCTCATCAGAAAGTCACAGCTCCCCCTCGCTCATCCACCAA------GCTCCAA
  Mordacia  TATAAAGGCGGCCGGAGAGGCGGAGAGAAGACACAACGACCTGTCAGCTCATCAGCAGCCTCAGCTCTCTTCGCTCTCAACCTTCAACCACCAGCTCCAG
   Geotria  TATAAAAGCAGCAGTC-AGACGGAGACAGAGCACAGCGACCTGACACCTTT-------CTCCCCAGCTCTCATCTCTCATCCAGCAAGCGCCCACTCCGA
            TATA box

            101                                                                                                200
Petromyzon  CTCCGATCACTATGGCCAGGTGAGTACACGCATA-GTCACAGTTA-CACACGCATCACACACACACACATAATCACAGTTAGACACAACAACAACCGCCA
  Mordacia  CTCCATCCGCAATGGCCAGGTGCGTCCACGCAGGTGTCGCAGTGCGCGCATGCAGCGCACGCACGCACGCATACAGTCACA---------------
   Geotria  CT-----CGCAATGGCCAGGTGAGTAGATGCAT-----------------GCCACATTCACACACACACACTCAATCACACACAACT-------------
            exon 1      start    intron

            Note: The sequence alignment is not shown over the entire length of the intron.

            2001                                                                                              2100
Petromyzon  GCTCTACCTTTGACGGGAGGAGCCCTTTGGGAATTCACCCCCTCGATGTTGTTTTTGTATTCCCCCCCCCCACCGCTCTACTGCTCGCAGCTGCCCAGAC
  Mordacia  --------------GCGAGCTCCCCTTAAGG-----------------------TGGAATTAACTCTCGTGCGTTGCCCGATGCCCGCAGTTGCCCAGAC
   Geotria  -------------------------------------------------------------------------CTGTCGTATTGCCTCAGCTGCCCAGAC
                                                                                                  intron  exon 2
            2101                                                                                              2200
Petromyzon  ACGACCCCCACCATGGAGATGACCGCCGAGAACCAGGAGGCCGCTGGTGAAGCCTGCGGCCGCAGCTGC---TACGGCCACGGAGGCTACGGCCACGGAG
  Mordacia  ACGACCCCGGCCATGGAGATGAGCGCCGAGAACCAGGATTCGTCCGCCGAGGCGTGTGGCCGCAGCTGCGGCTACGGCCACGGAGGCTACGGCCACGG--
   Geotria  ACCACCCCAACCATGGAGATGAGCGCCGACAACCAGGAGGCCGCCGGCGAGGCGTGCGGCCGTAGCTGC------GGCCACGGAGGCCACGGCTACGGAG

            2201                                                                                              2300
Petromyzon  GCTACGGCCACGGGGGTTACGGCCACGGA------------------------------------GGCTATGGCCACGGAGGCTATGGCCATGGAGGCTA
  Mordacia  -CTACGGCCACGGGGGCTACGGCCACGGCTACGGCCACGGAGGCTACGGCCAC------------GGCTACGGCCACGGAGGCTACGGCCACGGGGGCTA
   Geotria  GATACGGCCAAGGGGGATACGGCCACGGA---GGCTACGGAGGCTACGGGCACGGAGGCTACGGGGGCTACGGGCACGGAGGCTACGGGCACGGAGGCTA

            2301                                                                                              2400
Petromyzon  TGGCCACGGAGGC---------TATGGCCACGGAGGCTATGGCCACGGAGGCTATGGCCACGGAGGCTATGGCCATGGAGGCTACGGCCACGGAGGCTAC
  Mordacia  CGGCCACGGAGGC---------TACGGCCACGGAGGCTACGGCCACGGGGGCTACGGCCACGGAGGCTACGGCCACGGAGGCTACGGCCACGGGGGCTAC
   Geotria  CGGCCACGGTGGCCACGGTGGCTACGGCCACGGAGGCTACGGGCACGGAGGCTACGGCCACGGGGGCTACGGCCACGGAGGCTACGGCCACGGGGGCCAG

            2401                                                                                              2500
Petromyzon  GGCCAAGCTCTGGGCTACGGCCACCACGGCTACGGCGGCTACGGCCTGGGCCACGGCCACCTGAGCACCTACACGCACGCGCAGGGCCACTACGCGTACC
  Mordacia  GGGCAAGCTCTGGGCTACGGCCACCACGGCTACGGCGGCTACGGGCTGGGTCACGGCCACCTGTGCACGTACACCCACGGGCAAGGCCACTACGCGTTCC
   Geotria  GGCACCGGCCTAGGCTACGGCCACCACGGCCACGGAGGCTACGGGCTGGGCCACGGCCACCTGTGCACCTACACGCACGCCCAGGGTCACTACGCCTTCC

            2501                                                                                              2600
Petromyzon  CGGCCAAGGGCTGCGTCTCGCCGTGCGCCCGCGGCGCTCCA-----------------------------------------------------------
  Mordacia  CGGCCAAGGGCTGCGCCAGCCCGTGCGCTCCCAAGTGCGCCACCCCGTGCCACCCGGC---------------------GCCCAAGCCGTGCTTCCCAGC
   Geotria  CCTCCAAGGGCTGCTCCAGCCCTTGCGCCCCCAAGTGCCCAGCCCGTGCGTCGTGCCCAAGTGCCCCAGCCCGTGCCCGCCCAAGTCGTGCTGCCCGAG

            2601                                                                                              2700
Petromyzon  --AGTCGCCGTGCTGCACCCCTTGCCCGCCGTGCGTGCCCAAGAAGTGCGAGCCGTGCCCGGTCACCAAGTTCAAGGGTGCCCCTCTCCCTGCCGCACC
  Mordacia  CAAGTCCGCGTGCTGCGCCCCGTGCCCGCCCTGCGTGCCCAAGAAGTGCGAGCCGTGCCCGGTCACCAAGTACAAGAGCTGCCCGTCTCCGTGCCATACC
   Geotria  CAAGGCCCCGTGCTGCCTGCCGTGCCCGCCTTGCCTGCCCAAGAAATGCGAGCCGTGCCCGGTCACCAAGTACAAGGGCTGCCCCTCTCCCTGCCACACC

            2701                                                 2757
Petromyzon  GTTGTCGCCGGAGGCTATGGCGGCTACGGAGGCTACGGCTACGCGGGCAAATACTGA
  Mordacia  GGCGCCG------GGTTCGGGATACACGGCGGTTACGGATACGCCGGTAAATACTAG
   Geotria  GGGTTTG---------------------GCGGGTACGGGTATGCCGGCAAATACTAG
                                                                 stop
```

Figure S1. Conservation of *KRTAPL1* gene structure and nucleotide sequence in three species of lampreys. The nucleotide sequences of the *KRTAPL1* gene of three species of lampreys were aligned. Red fonts indicate identity of nucleotides in all species. The sequences begin with the TATA box in the promoter and end with the stop codon. The coding sequence is highlighted by yellow shading. The sequence of the intron, which is shown only close to the border of the exons, is highlighted by blue shading. Species: sea lamprey (*Petromyzon marinus*), Australian lamprey (*Mordacia mordax*) and pouched lamprey (*Geotria australis*). GenBank accession numbers of nucleotide sequences: *Petromyzon*, NC_046133.1:3269195-3271835; *Mordacia*, JBCLOA010364153.1: 423-2155; *Geotria*, JAZEWU010000003.1: 17856448-17857950.

a

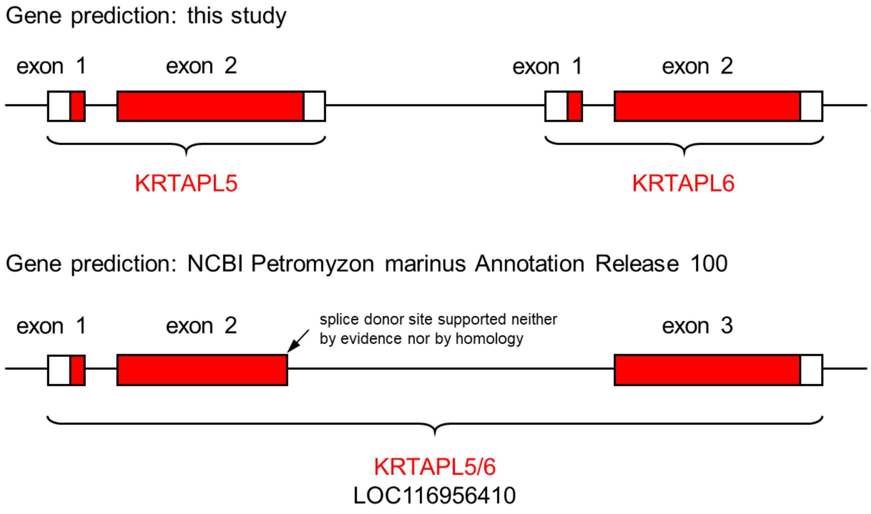

b

>Pm_KRTAPL5
MASFESATGEDCSSCHRRGGSCHHCGGRGCGHCRRLGHGLLGHGGYSQSCHGGYGIGGHLYGGHAGYGHGGYGTGGYGHGGYGHGGYGHGG
YGHGGYGHGGYGHGGYGHGGYGTGCCKPVTTCHTRPIVHLDFCTSGHGHSSCHHCGGRGCGHCGSGHFGGYGHY

>Pm_KRTAPL6
MASYECTADCHDECATSSCHHCGGKGCGHCRRRQSCHHCGGKGCGHCRRSSCHHCGGKGCGHCRRSSCHGFECDEEVGCGLTVGRSSCHRC
GGIGCGHCRRSSCHHCGGVGCGHCRRSSCHHCGGVGCGHCRRSSCHGFECDEEVGCGLSVGRSSCHHCGGKGCGHCRRSSCHHCGGKGCGH
CRRRQSCHHCGGKGCGHCRRSSCHGFECDEEVGCGLTVGRSSCHHCGGVGCGHCRRSSCHHCGGVGCGHCRRSSCHHCGGVGCGHCRRSSC
HRCGGVGCGHCRRQSCHHCGGKGCGVCRGWGIGGRKF

>Pm_KRTAPL5/6 GenBank prediction: XP_032833892.1
MASFESATGEDCSSCHRRGGSCHHCGGRGCGHCRRLGHGLLGHGGYSQSCHGGYGIGGHLYGGHAGYGHGGYGTGGYGHGGYGHGGYGHGG
YGHGGYGHGGYGHGGYGHGGYGTGCCKPVTTCHTRPIVHLDFCTSGHGHSSCHHCGGRGCGHCGSGHFGGYECTADCHDECATSSCHHCGG
KGCGHCRRRQSCHHCGGKGCGHCRRSSCHHCGGKGCGHCRRSSCHGFECDEEVGCGLTVGRSSCHRCGGIGCGHCRRSSCHHCGGVGCGHC
RRSSCHHCGGVGCGHCRRSSCHGFECDEEVGCGLSVGRSSCHHCGGKGCGHCRRSSCHHCGGKGCGHCRRRQSCHHCGGKGCGHCRRSSCH
GFECDEEVGCGLTVGRSSCHHCGGVGCGHCRRSSCHHCGGVGCGHCRRSSCHHCGGVGCGHCRRSSCHRCGGVGCGHCRRQSCHHCGGKGC
GVCRGWGIGGRKF

Figure S2. Prediction of *KRTAPL5* and *KRTAPL6* genes in comparison to *LOC116956410*. (a) Gene predictions of this study and in NCBI GenBank Petromyzon marinus Annotation Release 100. Note that the gene organization of the genes predicted in this study is homologous to the structure of the other *KRTAPL* genes of the sea lamprey (*Petromyzon marinus*, Pm). *KRTAPL5* and *KRTAPL6* are located on *Petromyzon marinus* isolate kPetMar1 chromosome 65, kPetMar1.pri, whole genome shotgun sequence, GenBank accession number NC_046133.1 at the following nucleotide positions: *KRTAPL5*, exon 1: 3319677-3319684, exon 2: 3321209-3321698; *KRTAPL6*, exon 1: 3340696-3340703, exon 2: 3343923-3344847. **(b)** Amino acid sequences of KRTAPL5, KRTAPL6 and KRTAPL5/6 derived by translation of the prediction LOC116956410. Cysteine (C) and tyrosine (Y) residues are highlighted by yellow and red shading, respectively. Glycine (G) residues and histidine (H) are highlighted by grey and magenta shading, respectively. Underlines mark sequences that were detected by the proteomic analysis of horny teeth. Carboxy-terminal residues (R, arginine; K, lysine) of tryptic peptides are indicated by bold fonts.

a

Petromyzon marinus KRTAPL1

```
MASCPDTTPTMEMTAENQEAAGEACGRSC

YGHGG
YGHGG
YGHGG
YGHGG
YGHGG
YGHGG
YGHGG
YGHGG
YGHGG
YGHGG
YGHGG
YGHGG

YGQALGYG

GY
GL
GH
GH

LSTYTHAQGHYAYPA

KGCVSPCAPRRSKSPCCTPCPPCVP
KKC-EPCPVTKFK-GCPSPCRTVVA

GGY
GGY
GGY
GYA
GKY
```

b

Petromyzon marinus KRTAPL6

```
MASYECTADCHDECATS

SCHHCGGKGCGHCRRRQ
SCHHCGGKGCGHCRRS
SCHHCGGKGCGHCRRSSCHGFECDEEVGCGLTVGRS
SCHRCGGIGCGHCRRS
SCHHCGGVGCGHCRRS
SCHHCGGVGCGHCRRSSCHGFECDEEVGCGLSVGRS
SCHHCGGKGCGHCRRS
SCHHCGGKGCGHCRRRQ
SCHHCGGKGCGHCRRSSCHGFECDEEVGCGLTVGRS
SCHHCGGVGCGHCRRS
SCHHCGGVGCGHCRRS
SCHHCGGVGCGHCRRS
SCHRCGGVGCGHCRRQ
SCHHCGGKGCGVCRGW

GIGGRKF
```

Figure S3. Lamprey KRTAP-like proteins contain sequence repeats. Amino acid sequences of lamprey KRTAPL1 **(a)** and KRTAPL6 **(b)** are shown with line breaks to align internal sequence repeats. Residues are color-coded as in Figure 3.
